# The critical need for an open medical imaging database in Japan: implications for global health and AI development

**DOI:** 10.1007/s11604-024-01716-y

**Published:** 2024-12-13

**Authors:** Daiju Ueda, Shannon Walston, Hirotaka Takita, Yasuhito Mitsuyama, Yukio Miki

**Affiliations:** 1 Department of Artificial Intelligence, Graduate School of Medicine, Osaka Metropolitan University, Abeno-ku, Osaka, Japan; 2 Department of Diagnostic and Interventional Radiology, Graduate School of Medicine, Osaka Metropolitan University, Abeno-ku, Osaka, Japan

**Keywords:** Open database, Artificial intelligence, Medical AI, AI fairness

## Abstract

Japan leads OECD countries in medical imaging technology deployment but lacks open, large-scale medical imaging databases crucial for AI development. While Japan maintains extensive repositories, access restrictions limit their research utility, contrasting with open databases like the US Cancer Imaging Archive and UK Biobank. The 2018 Next Generation Medical Infrastructure Act attempted to address this through new data-sharing frameworks, but implementation has been limited by strict privacy regulations and institutional resistance. This data gap risks compromising AI system performance for Japanese patients and limits global medical AI advancement. The solution lies not in developing individual AI models, but in democratizing access to well-curated Japanese medical imaging data. By implementing privacy-preserving techniques and streamlining regulatory processes, Japan could enhance domestic healthcare outcomes while contributing to more robust global AI models, ultimately reclaiming its position as a leader in medical innovation.

## Introduction

Japan has long been at the forefront of medical imaging technology, with its advanced diagnostic equipment being utilized in healthcare facilities worldwide. According to the Organization for Economic Co-operation and Development (OECD), Japan leads all member countries in the number of MRI and CT scanners per capita [1]. This technological advantage, however, stands in stark contrast to a significant shortcoming in Japan's contribution to global medical AI: the lack of open, large-scale medical imaging databases. The importance of such databases cannot be overstated in the current era of rapid AI advancement [2]. They serve as the foundation for developing and refining AI algorithms, which are increasingly becoming integral to medical diagnosis, treatment planning, and prognosis prediction [3]. The absence of Japanese data in these global AI training sets not only hinders Japan's potential to lead in medical AI but also has far-reaching implications for global healthcare advancements and AI fairness [4, 5].

## Current state of medical imaging data in Japan

While Japan possesses vast repositories of high-quality medical imaging data, there is a notable absence of what could be termed "big data" in an open, accessible format. The Japan Medical Imaging Database (J-MID) represents Japan's impressive collection of medical imaging data [6]. However, despite this valuable resource, Japan currently lacks a truly open, large-scale medical imaging database. While J-MID maintains a vast collection, its access restrictions prevent it from fully serving the broader research community. This situation stands in contrast to other nations where unrestricted medical databases have become powerful catalysts for advancing AI innovation and research. For instance, the Cancer Imaging Archive (TCIA) in the United States provides researchers with access to large volumes of de-identified medical images of cancer patients [7]. Similarly, the UK Biobank offers researchers access to medical imaging data from hundreds of thousands of participants [8]. These open databases have significantly accelerated AI research in both their respective countries and globally.

The hesitancy toward open data sharing in Japan reflects cultural and institutional patterns. Unlike Western healthcare systems that have increasingly embraced data sharing as a driver of innovation, Japan's medical institutions traditionally view patient data as institutional property to be carefully guarded. This perspective stems from a historically paternalistic medical system and cultural emphasis on privacy protection. Moreover, Japan's post-war development model, which emphasized adapting existing technologies rather than pioneering new approaches, might have created an institutional culture that can be hesitant to lead in data-sharing initiatives.

The Japanese government has attempted to address this issue through the Next Generation Medical Infrastructure Act, enacted in 2018 and revised in 2024, and established new frameworks for medical data utilization [9, 10]. The 2024 revision introduced 'partially de-identified medical information' categories and guidelines for clinical trials and linkage analysis with the National Database of Health Insurance Claims. This legislation aimed to promote the utilization of medical data while protecting patient privacy. However, its implementation has faced several challenges. As of 2024, only a handful of medical institutions have been certified under this act to provide anonymized medical imaging [11]. The stringent requirements for data anonymization have proved to be a significant hurdle for many institutions. Moreover, there is a lack of public understanding about the importance of data sharing for medical research, leading to hesitancy in participation.

To address the difficulties of collecting and utilizing medical data, a working group was convened in 2022. This working group published the Guidelines on the Utilization of Medical Digital Data for AI Research and Development in 2024 [12]. Acknowledging the importance of collaborations between academic institutions and private companies, the new guidelines provide a comprehensive framework for healthcare institutions and private companies to collaborate on AI medical device development. These guidelines aim to maintain a balance between innovation and patient privacy rights.

## Implications of the data gap

The scarcity of open Japanese medical imaging data has several critical implications for the overall robustness and generalizability of AI models. Recent studies have revealed troubling performance disparities in AI across different racial and gender groups [4, 13]. Seyyed-Kalantari et al. demonstrated that AI algorithms trained on chest radiographs showed lower performance for underrepresented patient populations [14]. With Japanese data largely absent from the training sets of global AI models, there is a real risk that these systems may underperform for Japanese patients, potentially compromising healthcare outcomes for Japanese citizens. Diversity in training data is crucial for developing AI systems that perform well across different populations [15]. By not contributing large-scale, open databases of Japanese medical imaging data, Japan is missing an opportunity to enhance the overall capabilities of global medical AI systems.

Recent advancements in Large Language Models (LLMs) [16–25] have further underscored the importance of this data gap. The integration of visual information and textual descriptions has become a cornerstone of cutting-edge medical AI applications [26, 27]. From automated diagnosis to personalized treatment planning and AI-assisted medical education, the ability to train models on paired image and language data is paramount. Japan's lack of such openly available datasets puts it at a significant disadvantage in this rapidly evolving field.

## Challenges in creating open databases

Several factors contribute to the difficulty in establishing open medical imaging databases in Japan. Japan has stringent privacy laws and cultural norms that prioritize individual privacy [28, 29]. The Personal Information Protection Act, amended in 2020, sets strict guidelines for handling personal data, including medical information [30, 31]. While these protections are crucial, they can also create barriers to data sharing and open database creation. Many medical institutions in Japan are reluctant to share their data, primarily due to concerns over potential data misuse, such as breaches of privacy or unethical use, and the extensive effort required to prepare data for sharing. Data preparation involves several time-consuming tasks, such as anonymization, ensuring compliance with legal and ethical standards, and addressing compatibility issues across different systems. To overcome these challenges, it is necessary to establish appropriate incentives, such as financial support, recognition for contributing to research, or access to shared resources, to motivate institutions to participate in data sharing. This institutional inertia is a significant obstacle to creating comprehensive, open databases. Developing the necessary infrastructure for secure, efficient data sharing and management is a complex and costly undertaking. It requires not only technical expertise but also ongoing maintenance and updates to ensure data integrity and security [32]. While the Next Generation Medical Infrastructure Act was intended to facilitate medical data utilization, its implementation has revealed several regulatory challenges. The complex certification process for institutions and the strict anonymization requirements have limited its effectiveness.

## Potential solutions and path forward

Addressing the challenges outlined above requires a multi-faceted approach. Implementing advanced anonymization and privacy-preserving techniques can help alleviate privacy concerns. Techniques, such as differential privacy, federated learning, defacing, and secure multi-party computation, can allow for data utilization while maintaining individual privacy [33]. Developing incentive structures for medical institutions to share their data could help overcome institutional resistance. This could include financial incentives, academic recognition, or priority access to developed AI tools [34]. Launching comprehensive public awareness campaigns is essential, particularly to clarify the personal information used, the value of utilizing this personal medical data in healthcare AI research, and the protective measures already in place for medical data sharing [35]. Such initiatives could help address common misconceptions about data misuse and build trust in medical AI development. Engaging in international collaborations and adopting global best practices in data sharing and AI development can help Japan align with the international standards while contributing its unique datasets to global research efforts [36]. Streamlining the regulatory process for data sharing and clarifying guidelines for anonymization and data usage could facilitate the creation of open databases while maintaining necessary protections.

## All we need is the open dataset

The advancement of medical AI does not solely depend on developing sophisticated algorithms or individual models—it hinges critically on the availability of comprehensive, high-quality datasets. While creating specific AI solutions for medical challenges is valuable, the most transformative approach may lie in democratizing access to well-curated medical imaging data. When researchers worldwide face a particular medical challenge, the most effective catalyst for innovation often is not developing a single AI solution, but rather publishing diverse, representative datasets that encompass that challenge [7, 37]. The open dataset approach creates a multiplier effect, where hundreds of researchers can simultaneously work on solutions, leading to rapid iterations and improvements that would not be possible with siloed development.

The success of projects like ImageNet in computer vision and the Cancer Imaging Archive in medical imaging demonstrates how open datasets can accelerate scientific progress exponentially [7]. By making both data and associated code publicly available, we create an ecosystem where researchers can build upon each other's work, validate findings across different populations, and identify potential biases or limitations. This collaborative approach is particularly crucial in healthcare, where diverse patient populations and varying clinical contexts require robust solutions that can only emerge from extensive testing and refinement across multiple research groups [4].

## Potential impact of open Japanese medical imaging databases

The establishment of open, large-scale medical imaging databases in Japan could have a far-reaching impact. By including Japanese data in AI training sets, models could be developed that perform more accurately and fairly for Japanese patients, potentially leading to better health outcomes [38]. The inclusion of diverse Japanese data in global datasets would contribute to the development of more robust and generalizable AI models, benefiting patients worldwide [39]. Fostering an environment conducive to medical AI research and development could attract international investment, stimulate domestic innovation, and potentially create new export opportunities in AI-driven healthcare solutions [40]. By taking a proactive role in medical data sharing and AI development, Japan could position itself as a leader in shaping international standards and ethical guidelines for AI in healthcare [41].

## Conclusion

The establishment of open, large-scale medical imaging databases in Japan represents both a national imperative and a global necessity. It offers an opportunity for Japan to leverage its strengths in medical imaging technology, contribute meaningfully to the advancement of AI in healthcare, and ensure better health outcomes for its citizens and people worldwide. The path forward requires collaboration between government, healthcare institutions, and the tech industry. While challenges remain, particularly in the areas of privacy protection and institutional buy-in, the potential rewards—in terms of improved health outcomes, scientific advancement, and economic opportunity—make this an investment in the future that Japan cannot afford to overlook. By taking bold steps to open its vast medical imaging resources to the global research community, Japan can reclaim its position as a leader in medical innovation and play a pivotal role in shaping the future of global healthcare. The time for action is now, as the rapid pace of AI development in healthcare makes the need for diverse, comprehensive datasets more pressing than ever.
